# Valuing natural habitats for enhancing coastal resilience: Wetlands reduce property damage from storm surge and sea level rise

**DOI:** 10.1371/journal.pone.0226275

**Published:** 2020-01-15

**Authors:** Ali Mohammad Rezaie, Jarrod Loerzel, Celso M. Ferreira

**Affiliations:** 1 Civil, Environmental, and Infrastructure Engineering, George Mason University, Fairfax, Virginia, United States of America; 2 CSS, Inc., under contract for NOAA National Centers for Coastal Ocean Science, Hollings Marine Laboratory, Charleston, SC, United States of America; Universidade de Aveiro, PORTUGAL

## Abstract

Storm surge and sea level rise (SLR) are affecting coastal communities, properties, and ecosystems. While coastal ecosystems, such as wetlands and marshes, have the capacity to reduce the impacts of storm surge and coastal flooding, the increasing rate of SLR can induce the transformation and migration of these natural habitats. In this study, we combined coastal storm surge modeling and economic analysis to evaluate the role of natural habitats in coastal flood protection. We focused on a selected cross-section of three coastal counties in New Jersey adjacent to the Jacques Cousteau National Estuarine Research Reserve (JCNERR) that is protected by wetlands and marshes. The coupled coastal hydrodynamic and wave models, ADCIRC+SWAN, were applied to simulate flooding from historical and synthetic storms in the Mid-Atlantic US for current and future SLR scenarios. The Sea Level Affecting Marshes Model (SLAMM) was used to project the potential migration and habitat transformation in coastal marshes due to SLR in the year 2050. Furthermore, a counterfactual land cover approach, in which marshes are converted to open water in the model, was implemented for each storm scenario in the present and the future to estimate the amount of flooding that is avoided due to the presence of natural habitats and the subsequent reduction in residential property damage. The results indicate that this salt marshes can reduce up to 14% of both the flood depth and property damage during relatively low intensity storm events, demonstrating the efficacy of natural flood protection for recurrent storm events. Monetarily, this translates to the avoidance of up to $13.1 and $32.1 million in residential property damage in the selected coastal counties during the ‘50-year storm’ simulation and hurricane Sandy under current sea level conditions, and in the year ‘2050 SLR scenario’, respectively. This research suggests that protecting and preserving natural habitats can contribute to enhance coastal resilience.

## Introduction

Coastal flooding from storm surge is a major concern for coastal communities worldwide, and is expected to increase with potential changes in storm climatology [1] and projected sea level rise (SLR) [2–7]. The combined impacts of storm surge and SLR are expected to exacerbate the existing flood protection [8] and amplify property damages in the coastal areas [9]. SLR is also likely to affect natural habitats in coastal areas, such as wetlands and marshes, through changes in tidal range, salinity, and sediment supply [10–12]. The global loss in coastal wetlands may reach 44% by 2080 due to one meter of SLR and human interventions for residential purpose [13], or as high as 78% by 2100, with 1.1 meters of SLR and maximum coastal dike construction [14]. The loss in coastal wetlands may worsen the issue of coastal flooding, as these natural habitats can reduce the impacts of flooding by attenuating storm surge [15–18], mitigating wave energy [19–21], and stabilizing shorelines [22]. However, the capacity to reduce flooding impacts varies with the coastal landscape [15], vegetation characteristics [23,24], and soil properties [25].

The flood protection services are considered to have indirect use values [26], or benefits derived from ecosystems through supporting and protecting activities [27]. These values depend on several factors, including flood reduction capacities of the habitats [28], the surrounding population [29], and property values [23]. Several methods can be used to estimate flood protection service values, including choice experiments [30], replacement cost [31], and avoided cost [23,28,32]. When assessing these protective services, it is important to address the complex interactions between storm surges and natural habitats [23], and incorporate the non-linear nature of the services [33,34]. For example, Barbier et al [23] captured the spatial variability of coastal landscape and storm surge flooding using a coastal hydrodynamic model to estimate the marginal value of wetlands flood protection services. Although the study were conducted on a single coastal transect using only hypothetical storms, the findings showed that 1% increase in coastal wetlands in Louisiana could reduce property damages by $99 to $133 for each sub-planning unit (consists of 1,780 households per sub-planning units). A recent study by Narayan et al. [35] also used high-resolution flood and loss models for 2,000 synthetic storm events to quantify the impacts of coastal wetlands on local annual flood losses in Barnegat Bay in Ocean County, New Jersey. The results of the study suggest that marsh can reduce property flood losses by an average of 16% annually. However, one limitation of their study is that all properties were assumed to be uniformly distributed and identically valued throughout the study area.

The goal of our study was to estimate the value of flood protection services provided by protected coastal wetlands and marshes adjacent to the Jacques Cousteau National Estuarine Research Reserve (JCNERR) to a cross-section of three coastal counties in New Jersey. Additionally, the study aims to integrate the natural variability of coastal storm surge and impacts of SLR on the natural habitats into the economic valuation of the flood protection services. The coupled version of the Advanced Circulation model (ADCIRC) and Simulating Waves Nearshore model (SWAN) was applied to compute the storm surge flooding for historical and synthetic storms. The Sea Level Affecting Marshes Model (SLAMM) was used to simulate the potential changes in the natural habitats, especially coastal wetlands and marshes, due to SLR. The simulated flood depths were combined with publicly available parcel-level property data to compute flood damages for the selected storms in both current and future conditions. In order to quantify the flood protection services, the reduction in damages due to the presence of the natural habitats are estimated based on the value of protected residential properties.

Coastal flooding from storm surge and SLR are already affecting coastal communities and ecosystems. Therefore, it is important to develop ecosystem-based flood protection approaches that will not only protect these communities, but also the ecosystems that can reduce the impacts of coastal flooding. In order to implement these approaches, it is essential to quantify the capacity and value of these natural habitats to protect communities from recurrent coastal flooding. In addition, it is essential to work closely with the coastal planners and natural resources area managers to integrate scientific solutions into effective ecosystem management plans for the reservation and conservation of the natural habitats [36]. This study improves upon the existing literature in three ways. First, it uses parcel-level property values when estimating avoided damages, which allowed for more spatially explicit results. Additionally, it accounted for storm wind-driven wave effects, as wave setup can significantly contribute to the increase in water levels due to hurricanes [37–39]. A recent study [39] suggested that wave setup contributed to 17% of the peak water level during most of the hurricane events during 1988–2015 in the US coast. Second, this study incorporated SLR and its impacts on coastal wetlands and marshes to provide a more realistic evaluation of the flood protection service in future scenarios. Finally, in this study, an interdisciplinary team of coastal engineers, marine and social scientists collaborated with natural resources managers of the JC NERR to provide further insights on valuing natural habitats for coastal resilience and support natural habitats restoration initiatives.

## Materials and methods

### Study area and Jacques Cousteau National Estuarine Research Reserve

**Fig 1:**
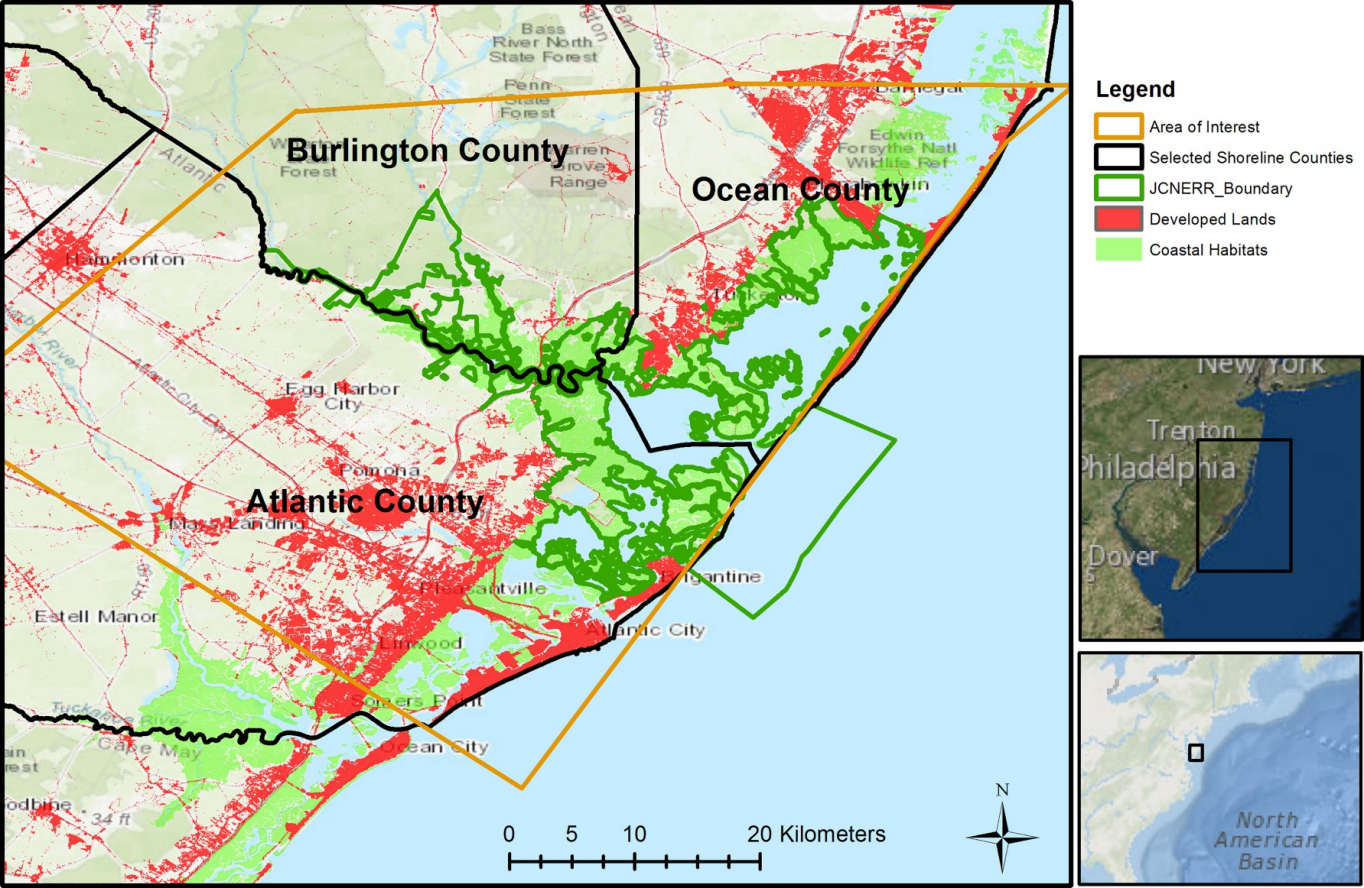
Study area, including locations of developed lands and natural habitats. (Base map source: ESRI [42–44]).

The study area (Fig 1) includes the JCNERR and portions of three surrounding counties: Ocean County, Burlington County, and Atlantic County. The JCNERR is a protected area with more than 45,000 hectares of terrestrial and estuarine ecosystems [40]. It is located along the southeastern coast of New Jersey, encompassing the lower Barnegat Bay, Little Egg Harbor, Great Bay, and inland back-bays extending up to nine kilometers downstream along the adjacent continental shelf of the Atlantic Ocean. Coastal marshes cover more than 28% of the reserve area [41], where the dominant vegetation types are smooth cordgrass (*Spartina alterniflora*) and salt-meadow cordgrass (*S*. *patens*). Although less than 2% of the total area is currently developed, the adjacent counties are experiencing high development rates and have a densely populated urban corridor [41]. More than 50% of the JCNERR is bounded by open water habitats, making the study area vulnerable to coastal flood hazards from storm surge and SLR.

### Coupled storm surge and waves model

**Fig 2:**
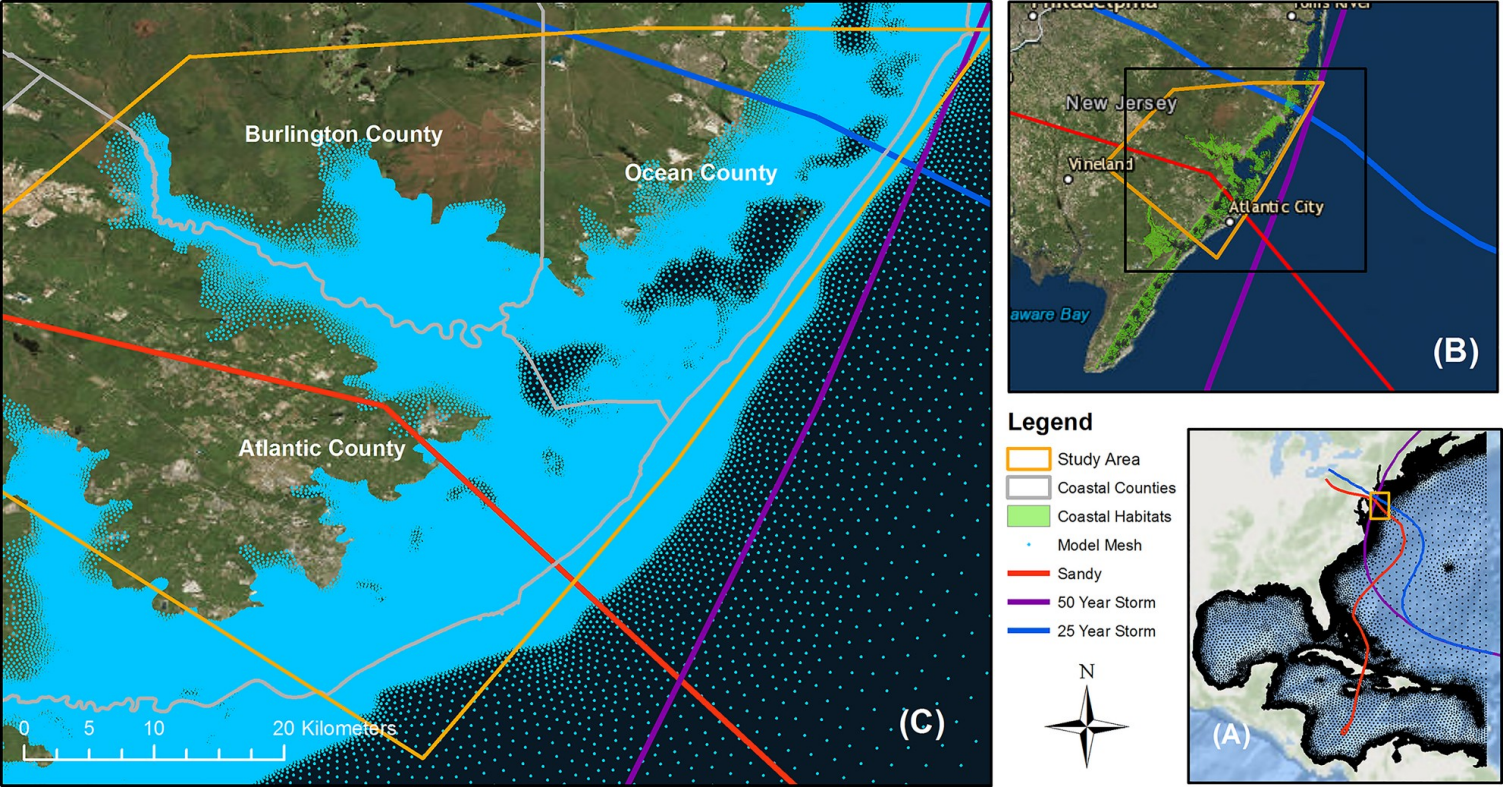
(A) Storm surge and waves model domain; (B) Selected storm tracks, (C) Mesh resolution in the study area (basemap source: ESRI [43,44]).

The coupled version of the coastal hydrodynamic model, ADCIRC, and the nearshore waves model, SWAN, was applied to the study area to simulate coastal flooding from storm surge and waves. ADCIRC is a widely used storm surge model [45–53] that solves shallow water equations to compute water surface elevation and vertically-integrated momentum equations to calculate current velocity [54,55]. The wind velocity and atmospheric pressure from hurricanes are calculated using the asymmetric hurricane vortex formulation embedded in ADCIRC to optimize computation of hurricane intensity in the model [56,57]. SWAN is a third generation wave model that uses the wave action balance equation to compute the full wave spectrum in the nearshore and offshore regions. In addition to allowing wave propagation from deep to shallow water areas, SWAN solves important nearshore wave processes, such as wave breaking, shoaling, refraction, and wind induced wave generation, in space and time [58]. In the coupled version, both models use the same computational mesh, model domain, and boundary. When coupled, ADCIRC computes water levels, currents, and wind information at each computational node to pass to SWAN, which calculates wave radiation stress and passes it back [59] to ADCIRC as a forcing function for the calculation in the next time step [38]. To estimate the flood depth, the validated Federal Emergency Management Agency (FEMA) Region II mesh [60,61] was used in the study area (Fig 2C). The unstructured mesh focuses on the Mid-Atlantic coastal counties of New Jersey and New York, and contains more than 0.6 million computational nodes with 30 to 500 meters of inland resolution [62]. Multiple national and regional topographic, shoreline, and bathymetry data sources were used to create a seamless digital elevation model (DEM) of ten meter resolution for developing the model elevation. The model domain, which extends from 60°W in the Atlantic Ocean and up to a 7.6 m inland contour are shown in Fig 2A. Further details about the mesh and terrain development are provided in the FEMA reports [61–63].

The current land use and land cover (LULC) information is collected from the Coastal Change Analysis Program (C-CAP) database [64] which represents the wetlands and marshes as estuarine and palustrine where both types are classified into three sub-categories such as forested, shrub and emergent wetlands. The land cover and land use, and their dissipation mechanism are represented in the model through frictional drag coefficients on the sea bottom and ocean surface. Manning’s roughness coefficient, or Manning’s N values are used to address shear stress by different LULC in the sea bottom while the free-surface shear stress is represented through Surface Canopy Coefficient and Surface Directional Effective Roughness Length. Based on the previous studies ([37,65–67] that used ADCIRC for storm surge simulation in similar coastal settings, the frictional coefficients are selected for this study. S1 Table shows the set of frictional parameters used in this study based on the LULC classification by C-CAP database which categorizes the wetlands and marshes as palustrine or estuarine types. Additionally, reduction in wind shear stress on the sea surface from vegetated canopies and other land cover is addressed using the Garret’s drag law [68]. More details on the dissipation mechanisms by LULC can be found in Westerink et al. [69], Ferreira et al. [65] and Atkinson et al. [66]. Although the representation of vegetated canopies through these frictional parameters, especially Manning’s *n*, is considered as a suitable practice for modeling storm surge in coastal landscape [15,35,69,70], they do not explicitly account for vegetation heights, density or diameter. It is also essential to include wave dissipation and attenuation by vegetation in the modeling approach [20,21] for better representation of the interactions between storm waves and wetlands. However, improvement of model parameters and processes is beyond the scope of our study. Further details about the model forcing and parameters are provided in S1 File.

The historical and synthetic storms were selected based on the recommendations by the protected area managers to capture variations in coastal flooding in the study area. Hurricane Sandy (2012), a 400-year storm event, was selected as the historical storm as it is currently the most catastrophic storm event in the Mid-Atlantic [71]. Two synthetic storms were selected from the US Army Corps of Engineers (USACE) North Atlantic Coast Comprehensive Study (NACCS) storm database. The NACCS developed an extensive database of synthetic storms based on observed records of storm surge and meteorological data for historical storms events along the US east coast from 1938 to 2013 [72]. Based on the storm surge and wave characteristics within the study area, these synthetic storms have a 4% (“25-year storm”) and 2% (“50-year storm”) respective probability of occurrence in any given year [72]. In relation to the study area, both Hurricane Sandy and the selected 25-year storm passed through the study area, whereas the selected 50-year storm traveled parallel to the shoreline (Fig 2B). The characteristics of the three storm events when passing the study area are summarized in Table 1.

**Table 1:** Characteristics and intensity of selected storms near the study area.

Storm Event	Min Central Pressure (mb)	Max Wind Speed (kt)	Max Sustained Wind Speed (kt)	Radius of Max Wind (nm)
Sandy	940	100	70	80
50-year storm	970	84	83	22
25-year storm	986	64	63	26

### Sea level rise and modeling marsh migration

SLAMM was applied to the study area to project potential changes in natural habitats due to SLR in the year 2050. The mid-century projection is selected based on the recommendations by the JCNERR managers for the restoration decisions. SLAMM uses coastal elevation, LULC, tidal and shoreline information, wave action, accretion rate, and the rate of SLR to model local changes in coastal ecosystems, such as wetland conversions and shoreline modification, from long term SLR [73–76]. It applies different threshold values for tidal range, salinity, accretion and proximity to shoreline from previous literature [75] to determine whether coastal marshes can sustain their elevation relative to sea level or if they will migrate inland, convert into other land cover, or submerge under the sea.

For the SLAMM model, the LULC data is collected from the United States Fish and Wildlife Service (USFWS) National Wetlands Inventory (NWI) [77]. For consistency in representing the projected LULC in ADCIRC+SWAN, the habitats classification from NWI was converted to C-CAP types using a database description report [75,78,79] and previous literature [80]. A 10 meter resolution DEM was prepared using the US Geological Survey (USGS) National Elevation Dataset [81], and tidal information was collected from the National Oceanic Atmospheric Administration (NOAA) tidal data for New Jersey [82] using the average high tides from two tidal stations for use in SLAMM. Due to the recommendations by the natural resources managers of the JC NERR for their habitat restoration initiatives, a 1.7 millimeters per year of SLR is used based on the International Panel on Climate Change (IPCC)’s Special Report on Emissions Scenarios A1F1 scenario [83–85]. Kopp et al. [86] also suggested a similar rate (1.3 ± 0.2 mm/yr) of SLR for the coastal region of New Jersey. Additionally, rate of accretion can vary with wetland types, sediment characteristics, tidal range etc.[87]. To the best of our knowledge, we have not found any literature that suggested specific rates of accretion for different types of wetlands, and sediment characteristics in our area of interest. Considering the inadequacy of previous literature on rates of accretion or subsidence in the study area, an average of 4 millimeters per year of accretion is applied in the model as it is the best accretion value given for the study area based on previous work done by NOAA’s Coastal Services Center [88]. Using the given SLR rate, emission scenario, land elevation, tidal range and accretion, SLAMM estimated a total 0.1332m of SLR in the year 2050 for our study area. The start date for the DEM and NWI in the SLAMM model is set to the year 2010. Thus, the study utilized available data and recommendations by natural resources managers of the JC NERR based on their natural habitats restoration initiatives to develop the future scenario. However, it should be noted that there are uncertainties and limitations in projecting both future SLR and coastal landscape or salt marshes response to the projected SLR. While SLAMM has been widely applied to estimate the potential impacts of SLR on coastal wetlands in the US [74,76,89,90], it is not the most robust modeling tool to predict marsh changes due to SLR. However, there are trade-offs between collecting long term place-based organic and morphodynamic data, and simulating marshes responses due to SLR [91]. Therefore, for this study, SLAMM is applied as it simulates the key processes related to wetlands conversion due to different rates of SLR in a large region using nominal computational time. Further details about the SLAMM model processes can be found in the technical documentation [75].

### Ecosystem service valuation

The damages avoided method [23,35,92] was applied to value the ecosystem service of shoreline protection to residential properties from natural habitats, specifically coastal wetlands and marshes. The method considers value of the protected properties as a measure of the flood protection services provided by the natural habitats. For each storm event and for both current and future LULC conditions, flood elevations for “habitat present” and “habitat absent” scenarios were simulated. In the “habitat absent” scenario, natural habitats were artificially replaced with open water in the ADCIRC+SWAN model. The estimated difference in residential flood damages between these two scenarios was used as the value of reduced, or avoided, damages provided by the natural habitats.

Flood damages were estimated using depth-damage functions from the USACE [93], parcel level property data from the New Jersey Department of Treasury [94], and mean flood depth from ADCIRC+SWAN. The simulated maximum flood elevation due to each storm is incorporated into ArcGIS using the ArcStormSurge [95] tool to create flood elevation raster for the study area. The flood depth is then calculated by subtracting the land elevation values (from a 10m resolution DEM for our area of interest) from the flood elevation raster. The damage-depth functions estimate the expected percentage of property damage for varying levels of flood depth and property characteristics, such as the property elevation, number of stories, and whether or not the property was split level. Properties were only included in the damage estimation if the parcel centroid intersected the flood depth raster [96,97].

This analysis focused on residential parcels and their associated “improvement” value, which was used as the parcel value [96] for calculating monetary damages. Improvement value refers to the value of “improvements” made to the land which is usually the building value. Of the 111,866 residential parcels, 92 were removed from the analysis because they had no information on assessed value. An additional 7,847 parcels were removed because they had no information on the number of stories or whether the building was split-level (inputs required for the depth-damage functions). This resulted in a total residential parcel population of 103,927 with the information required for analysis. The assessed building values of the parcel population ranged from $15 to $5, 690,000, with a mean of $151,898, and 80% of the buildings are valued at $200,000 or less. This wide range of values is expected given that the study area includes rural inland watershed homes as well as oceanfront homes on barrier islands.

Two additional assumptions were made for analysis: 1) if a parcel had a non-integer number of stories, then that number was rounded up, 2) the parcel data did not include information on the presence or absence of basements, so the more conservative depth-damage functions for properties without basements were used based on anecdotal evidence concerning the lack of basements in the study area provided by local partners (Phil Reed, pers. comm., 2017; Mike Fromosky, pers. comm., 2017).

## Results

### Potential changes in natural habitats from SLAMM

Results from SLAMM suggest the total marsh area, which includes transitional, regularly, and irregularly flooded salt marshes, would reduce by about 5.4% in the study area due to SLR and marsh migration (Table 2). For example, transitional salt marshes and irregularly flooded marshes are predicted to decrease by around 38% and 36%, respectively, and become regularly flooded salt marshes, which increase considerably in the future. Additionally, low tidal open spaces, such as tidal flats, are predicted to increase substantially, which suggests SLR would cause the wetlands and marshes to migrate inland and convert into tidal flat [12].

**Table 2:** SLAMM simulated land cover change within the study area.

Land Cover Type (SLAMM)	Tidal Category	Current Scenario (km^2^)	Future Scenario (km^2^)	Change (%)
Developed Dry Land	Non-Tidal	1,030.95	1,029.92	-0.10%
Swamp	Freshwater Non-Tidal	375.56	375.50	-0.02%
Cypress Swamp	Freshwater Non-Tidal	0.01	0.01	0.00%
Inland Fresh Marsh	Freshwater Non-Tidal	15.37	15.34	-0.23%
Tidal Fresh Marsh	Freshwater Tidal	0.82	0.78	-4.80%
Transitional Salt Marsh	Transitional	3.20	2.00	-37.67%
Regularly-flooded Marsh	Saltmarsh	23.54	88.18	274.65%
Mangrove	Transitional	0.02	0.02	-0.59%
Estuarine Beach	Low Tidal	2.65	2.69	1.28%
Tidal Flat	Low Tidal	0.53	13.71	2,506.14%
Ocean Beach	Low Tidal	4.93	5.00	1.31%
Inland Open Water	Open Water	21.17	18.96	-10.46%
Riverine Tidal	Open Water	2.73	1.19	-56.36%
Estuarine Open Water	Open Water	290.91	294.66	1.29%
Open Ocean	Open Water	135.90	135.90	0.00%
Irregularly Flooded Marsh	Transitional	212.54	137.10	-35.49%
Inland Shore	Freshwater Non-Tidal	0.72	0.72	0.00%
Tidal Swamp	Freshwater Tidal	16.56	16.43	-0.76%

### Reduction in flooding by natural habitats

According to the results from ADCIRC+SWAN, when natural habitats are present, up to 23.1% fewer residential parcels would be inundated under current conditions and up to 3.7% fewer under future conditions (Table 3). The presence of the natural habitats are also expected to reduce the mean parcel-level flood depths by a maximum of 4.8% and 13.9% under current and future conditions respectively. Additionally, the mean proportional property damages can decrease up to 4.4% under current conditions and up to 4.0% under future conditions due to the presence of the natural habitats. All of these rate of changes (percent change) are greatest under the 25-year storm event under current conditions and under the 50-year storm under future conditions.

**Table 3:** Estimated flood depth and property damage changes from each storm event under current and future scenarios.

	25-year storm			50-year storm			Sandy		
	Habitat Present	Habitat Absent	% Change	Habitat Present	Habitat Absent	% Change	Habitat Present	Habitat Absent	% Change
**Current Scenario**									
Number of Flooded Parcels	3,592	4,423*	*23*.*13%*	3,932	4,723*	*20*.*12%*	46,598	46,820	*0*.*48%*
Mean Parcel Level flood depth (m)	0.021	0.022	*4*.*76%*	0.029	0.03	*3*.*40%*	1.055	1.065*	*0*.*95%*
Percent of property damage (Mean)	11.4%	11.9%*	*4*.*39%*	11.6%	11.7%*	*0*.*86%*	32.3%	32.5%	*0*.*62%*
**2050 SLR Scenario**									
Number of Flooded Parcels	5,895	5,973	*1*.*32%*	15,903	16,490*	*3*.*69%*	46,577	47,017*	*0*.*94%*
Mean Parcel Level flood depth (m)	0.031	0.033	*6*.*45%*	0.202	0.230*	*13*.*86%*	1.226	1.246*	*1*.*63%*
Percent of property damage (Mean)	11.9%	12.0%	*0*.*84%*	15.0%	15.6%*	*4*.*00%*	35.3%	35.7%*	*1*.*13%*

*Statistically significant increase compared to habitat present scenario at the 95% confidence level

**Fig 3:**
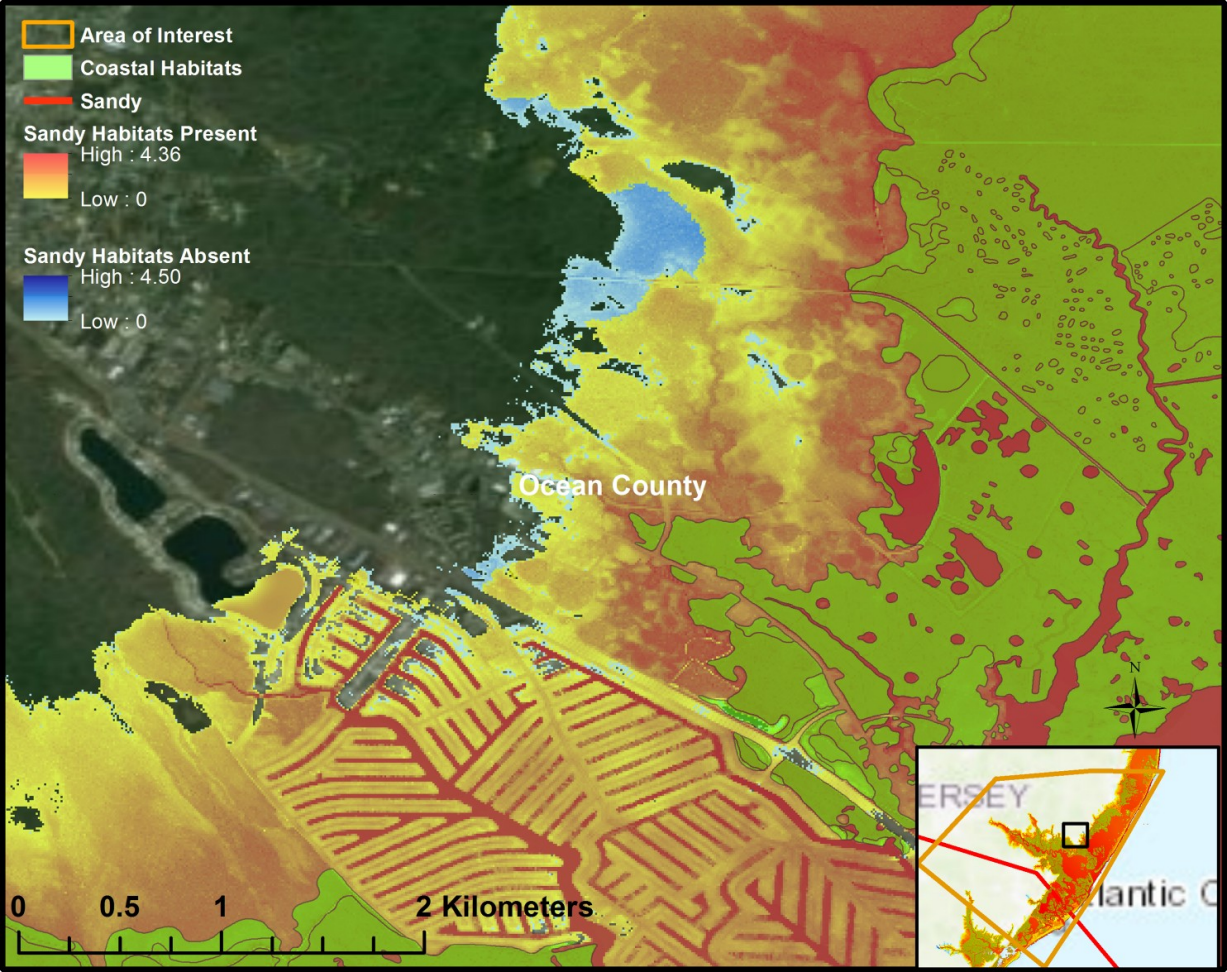
Reduction in flooding during Hurricane Sandy due to the presence of natural habitats (basemap source: ESRI [42,43]).

Our results also suggest that the presence of natural habitats can reduce the flood elevation in the shoreline areas. Fig 3 is an example output from the ADCIRC+SWAN model showing simulated flooding with and without habitat present under current conditions and a Hurricane Sandy storm event. Depending on the storm event and sea level rise conditions, presence coastal wetlands and marshes in the JCNERR can lower the flood elevation by a maximum of 0.2 to 0.4 meters. However, the averaged reduction in flood elevation and depth (elevation is the height above sea surface and depth is height of the inundation) are relatively low and varies from 0.03 to 0.06m.

### Flood protection value of coastal marshes

Results from the ecosystem service valuation suggest that presence of the natural habitats can reduce total property damage in the study area up to 13.8% under current conditions and up to 6.1% in future conditions considering the selected storm events (Table 4). The overall avoided damage by natural habitats during a single storm event can range from $8,500,000 to $13,000,000 under current conditions, while the value varies from $1,500,000 to $32,000,000 under 2050 conditions. The percentage changes are highest under the 50-year storm event for both temporal conditions. However, the flood protection value is highest in terms of absolute value for the 50-year storm and Hurricane Sandy under current conditions and 2050 conditions, respectively.

**Table 4:** Estimated parcel level property damages and avoided damages due to the presence of natural habitats.

	Property Damage (Habitat Present)	Property Damage (Habitat Absent)	Avoided Damage (Flood Protection Value)	Percent Change (From Habitat Present)
**Current Scenario**				
**25-year storm**	$82,062,657	$91,894,099	$9,831,442	11.98%
**50-year storm**	$94,888,388	$107,972,822	$13,084,434	13.79%
**Sandy**	$2,322,731,031	$2,331,067,963	$8,336,932	0.36%
**2050 SLR Scenario**				
**25-year storm**	$125,436,468	$126,980,226	$1,543,758	1.23%
**50-year storm**	$329,190,819	$349,122,514	$19,931,695	6.05%
**Sandy**	$2,562,559,835	$2,594,648,892	$32,089,057	1.25%

**Fig 4:**
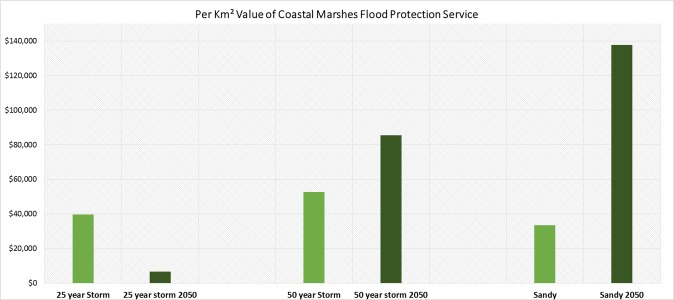
Estimated unit (km2) value of the flood protection services provided by natural habitats.

Additionally, when comparing per-area benefits, the predicted reduction in property damage due to the presence of natural habitats under future conditions is the highest during the Hurricane Sandy event (Fig 4). Results suggest that one square kilometer of natural habitats can reduce residential property damages in the study area from $34,000 to $53,000 per square kilometer under current conditions and from $7,000 to $138,000 per square kilometer under future conditions for the selected storm events. While the per-area value of protective services for both Sandy and the 50-year storm increased in the SLR scenario, the value decreased for the 25-year storm.

## Discussion

The goal of this study was to estimate the value of flood protection services provided by natural habitats and provide insights on how these services may alter with SLR. The place based analysis focused on the flood protection services by the coastal wetlands and marshes within and adjacent to the JCNERR to a cross-section of three coastal counties in New Jersey. The results show that the natural habitats can protect up to $13.1 and $32.1 million of residential property damage during the selected storm events in the study area in the current condition and the future scenario respectively, which suggests that the per square kilometer value of their flood protection services vary from a maximum of $53,000 to $138,000 under current and future conditions respectively. In all cases, our results indicate that the presence of wetlands and marshes reduces the property damage, though the total value varies largely by the storm and SLR conditions. These results could be due, in part, to the fact that each storm has unique characteristics from specific track, location, and intensity, and the variation in flood protection by natural habitats depends on storms track, forward speed and strength [15,69]. Moreover, the study area includes a range of residential types, from rural inland homes to oceanfront homes on the Barrier Island, where the building values vary substantially. This indicates that a parcel-scale analysis is necessary to capture the spatial variation of property values when assessing the flood protection services of coastal wetlands and marshes.

Our results suggests that the natural habitats can reduce total property damage from storm surge flooding by 6.1% to 13.8% (Table 4) within the study area, which are highest under the 50-year storm event for both temporal conditions. Although a maximum of 0.2 to 0.4m reduction in flood depth is found due to the presence of the natural habitats in the JCNERR, the mean reduction in parcel level flood depth show insignificant changes. Thus, the reduction in water level due to the presence of natural habitats largely vary with location, local elevation and geomorphologic features [16,17]. In terms of percent reduction in flood depth and property damage, however, the marsh showed higher potential in the 25-year and 50-year storms than it did in Hurricane Sandy. This indicates higher efficacy of natural flood protection during relatively frequent, low intensity storms. These results also support past literature [98] that demonstrated that there is a threshold effect where wetlands and marsh are predicted to be more protective under relatively more frequent and less intense storm events. Thus, natural habitats in coastal areas may provide substantial flood protection during recurrent flooding events and act as the first line of defense in more extreme storm events like Sandy. As global warming has the potential to increase storm intensity [99,100], and sea level rise can decrease total marsh area, the marginal value of natural habitats’ flood protection services may increase in future.

Additional results suggest that marsh migration and transition will occur due to SLR by 2050, leading to a loss of 5.4% of the total marsh area in the study area. For example, about one-third of the existing irregularly flooded and transitional marshes in the study area are predicted to decrease, whereas regularly flooded marsh is predicted to increase almost four-fold. This indicates the transition and migration of salt marshes, which can be hindered by anthropogenic interventions and socio-economic incentives, such as conversion of wetlands areas into developed lands, agriculture and aquaculture etc. [12,101]. Additionally, the area of tidal flats are predicted to increase nearly twenty-six-fold, which can affect the morphology of the natural habitat areas, as these tidal mud-flats are erosive during storm events [102]. This may impact the sustainability of the natural habitats since vegetation in the salt marshes can maintain their relative elevation to SLR through accumulating organic substance, trapping sediments [12], and reducing erosion [103]. Moreover, SLAMM model addresses eco-geomorphic feedbacks in the coastal marshes (which counteracts the impacts of SLR; [101] in a simplified manner which may overestimate their vulnerability to SLR [104].

One potential limitation of this study is the representation of wave energy dissipation by vegetation in modeling the interaction between storm surges and natural habitats. While the coupled version of the hydrodynamic and wave model addresses the loss in energy and momentum of combined surge and waves by vegetation features, the model does not account for different plant characteristics and their wave dissipation mechanisms. Although improvements in representing wave dissipation by vegetation can provide a more accurate quantification of reduction in surge and waves by vegetation in natural habitats, the implementation of more advanced wave reduction formulation in this or similar studies will require extensive field experimentation in different natural habitats under wide range of wave conditions and respective model validation. Furthermore, this study used the relative difference between two scenarios using the same model formulation to estimate the potential of natural habitats to relatively reduce flood depth. Thus, in our study, specific changes in model parameterization will not affect the analysis of reduction in water level due to the presence of natural habitats. A second limitation is that only three storm events were considered, and additional storm simulations would provide more robust and more generalizable results. The results also found large difference between maximum and average reduction in flood elevation due to presence of the natural habitats. Thus, our findings emphasize on primary data collection in coastal wetlands and marshes during storm events for improved quantification of their flood reduction capacity. Finally, the natural habitats provide several ecosystem services beyond flood protection. This study analyzed only the protection to residential structures, which suggests that these ecosystem service values are conservative estimates.

## Conclusion

The results of this study suggest that natural habitats can reduce both storm surge flood depth and property damage under current and future marsh conditions and under sea level rise. In particular, the natural habitats may offer better protection from relatively frequent, low intensity storms. These findings suggest that natural habitats can increase resilience in areas that are vulnerable to storm surge and sea level rise. In addition, the multidisciplinary framework presented in this study can assist natural resource managers and coastal landscape designers in the development of sustainable strategies to protect coastal communities, properties, and ecosystems from storm surge and sea level rise. As coastal flooding from storm surge has become a rising concern to coastal communities, it is important to develop ecosystem-based flood protection approaches that will protect both these communities and protective ecosystems. There is also need for improved understanding about the biophysical interactions between natural habitats, extreme flooding events, sea level rise, and the sustainability of these natural ecosystems in the future. This study advances the current understanding of coastal ecosystem service valuation by analyzing the role of natural habitats for flood protection and enhancing coastal resilience.

## Supporting information

S1 Table Frictional parameter values applied in the storm surge and waves model. (DOCX)

S1 File Additional description on model forcing and parameters. (DOCX)
