# Supplementary material for: Valuing natural habitats for enhancing coastal resilience: Wetlands reduce property damage from storm surge and sea level rise
Source: PLoS One. 2020 Jan 15;15(1):e0226275. doi: 10.1371/journal.pone.0226275 (PMC6961847; doi:10.1371/journal.pone.0226275)
Supplement: S1 Table — (DOCX) [file pone.0226275.s001.docx]

**S1 Table: Frictional parameter values applied in the storm surge and waves model.**

| Land Cover Type | Manning’s N | Surface Canopy Coefficient | Surface Directional Effective Roughness Length |
| --- | --- | --- | --- |
| Unclassified | 0 | 1 | 0 |
| Developed, High Intensity | 0.12 | 1 | 0.3 |
| Developed, Medium Intensity | 0.12 | 1 | 0.3 |
| Developed, Low Intensity | 0.07 | 1 | 0.3 |
| Developed, Open Space | 0.035 | 1 | 0.3 |
| Cultivated Crops | 0.1 | 1 | 0.06 |
| Pasture/Hay | 0.055 | 1 | 0.06 |
| Grassland/Herbaceous | 0.035 | 1 | 0.04 |
| Deciduous Forest | 0.16 | 0 | 0.65 |
| Evergreen Forest | 0.18 | 0 | 0.72 |
| Mixed Forest | 0.17 | 0 | 0.71 |
| Scrub/Shrub | 0.08 | 1 | 0.12 |
| Palustrine Forested Wetlands | 0.2 | 0 | 0.6 |
| Palustrine Scrub/Shrub Wetlands | 0.075 | 1 | 0.11 |
| Palustrine Emergent Wetlands | 0.07 | 1 | 0.3 |
| Estuarine Forested Wetlands | 0.15 | 0 | 0.55 |
| Estuarine Scrub/Shrub Wetlands | 0.07 | 1 | 0.12 |
| Estuarine Emergent Wetlands | 0.05 | 1 | 0.3 |
| Unconsolidated Shore | 0.03 | 1 | 0.09 |
| Barren Land | 0.03 | 1 | 0.05 |
| Open Water | 0.025 | 1 | 0.001 |
| Palustrine Aquatic Bed | 0.035 | 1 | 0.04 |
| Estuarine Aquatic Bed | 0.03 | 1 | 0.04 |
