# Supplementary material for: Valuing natural habitats for enhancing coastal resilience: Wetlands reduce property damage from storm surge and sea level rise
Source: PLoS One. 2020 Jan 15;15(1):e0226275. doi: 10.1371/journal.pone.0226275 (PMC6961847; doi:10.1371/journal.pone.0226275)
Supplement: S1 File — (DOCX) [file pone.0226275.s002.docx]

**Additional Description on Model Forcing and Parameters**

The model is forced with tides in the open ocean boundary and wind using harmonic tidal constituents from the Le Provost et al. 1994). It includes the major diurnal tidal constituents (K1, O1, and P1) and all of its semidiurnal constituents (M2, S2, N2, K2). The wind, pressure and track information for Hurricane Sandy is taken from the National Hurricane Center (NHC) Hurricane Data 2nd generation (HURDAT2) database while atmospheric forcing data for the Synthetic storms ( 25- and 50-year storm) are collected from the North Atlantic Coast Comprehensive Study (NACCS) storm database. We used the asymmetric hurricane vortex formulation (Mattocks et al 2006; Mattocks & Forbes 2008) based on the Holland wind model (Holland 1980) in ADCIRC to calculate wind velocity and atmospheric pressure from hurricanes at each node. Sea level rise is incorporated in the model by the eustatic method (Frey et al. 2010; Passeri et al. 2015, 2016; Bilskie et al. 2014) where the mean sea water level is offset by the SLR value in the model.
